# Metabolomic Insights into Head and Neck Cancer: Recent Advances and Future Directions

**DOI:** 10.3390/curroncol33040201

**Published:** 2026-03-31

**Authors:** Srikanth Ponneganti, Kousalya Lavudi, Maharshi Thalla, Gayatri Narkhede, Reva Dwivedi, Rekha Kokkanti, Prashant Pandey

**Affiliations:** 1 Department of Food Science and Technology, The Ohio State University, Columbus, OH 43210, USA; 2 Department of Radiation Oncology, The Ohio State University, Columbus, OH 43210, USA; 3 Comprehensive Cancer Center, The Ohio State University, Columbus, OH 43210, USA; 4 Department of Pharmaceutical Sciences, Irma Lerma Rangel School of Pharmacy, Texas A&M University, Kingsville, TX 78363, USA; 5 Biocon Bristol Myers Squibb Research & Development Center, Bengaluru 560099, Karnataka, India; 6 Department of Pharmaceutical Sciences, Chhatrapati Shahu Ji Maharaj University, Kanpur 208024, Uttar Pradesh, India; 7 School of Biotechnology, KIIT University, Bhubaneswar 751024, Odisha, India; 8 Department of Pharmaceutical Sciences, Babasaheb Bhimrao Ambedkar University, Lucknow 226025, Uttar Pradesh, India; 9 Faculty of Pharmacy and Pharmaceutical Sciences, University of Alberta, Edmonton, AB T6G 2E1, Canada

**Keywords:** squamous cell carcinoma, metabolomics, tumor microenvironment, cancer-associated fibroblasts

## Abstract

Head and neck squamous cell carcinoma (HNSCC) continues to pose a major global health challenge, with over 600,000 new cases diagnosed annually and persistently poor survival outcomes despite advances in surgery, radiotherapy, and immunotherapy. Growing evidence implicates metabolic reprogramming, including enhanced glycolysis, glutaminolysis, lipid synthesis, and one-carbon/redox flux as a central driver of HNC initiation, progression, and therapy resistance. In contrast, metabolic crosstalk within the hypoxic, acidic tumor microenvironment (TME) further shapes immune evasion and stromal support. Recent innovations in mass spectrometry platforms (LC-MS, GC-MS, NMR) have attracted attention in clinical therapeutics, and spatial metabolomics imaging techniques now enable high-resolution in situ mapping of metabolites, revealing intratumoral heterogeneity and offering new insights into tumor-immune–stromal interactions and potential biomarkers for precision oncology. In this review, we integrate critical methodological considerations from sample collection and data-analysis workflows to analytical pitfalls with a balanced, pathway-focused analysis of HNSCC dysmetabolism, examine tumor immune stromal metabolic interactions, and highlight translational opportunities through emerging biomarkers, targeted inhibitors, and cutting-edge approaches such as single-cell and AI-driven metabolomics to chart a roadmap toward precision oncology for HNSCC.

## Simple Summary

Head and neck cancer remains a significant global health challenge, often diagnosed too late for effective treatment. Cancer cells survive and grow by hijacking the body’s energy production processes, creating a unique chemical footprint. This review explores “metabolomics,” the study of these chemical markers, to understand how tumors fuel themselves and evade the immune system. We examine how advanced technologies can detect specific metabolic markers in readily accessible samples such as saliva and blood. These markers could serve as early warning signs or tools to monitor disease progression. By mapping these metabolic changes, scientists can develop targeted therapies that cut off the tumor’s energy supply or boost the immune response. Ultimately, these advances pave the way for “precision oncology,” offering non-invasive diagnostic options and personalized treatments to improve patient survival rates.

## 1. Introduction

Head and neck squamous cell carcinoma (HNSCC) ranks among the most common cancers worldwide and includes a diverse range of malignancies arising from the upper aerodigestive tract and originating from the mucosal epithelium of the oral cavity, pharynx, and larynx [1,2,3]. Of the above-mentioned, HNSCC accounts for almost 90% of head and neck cancers (HNC) and has an estimated global incidence of approximately 600,000 cases per year, representing about 7.6% of all cancers and 4.8% of all cancer-related deaths [1]. As per the 2024 statistics, considering the oral cavity and pharynx, tongue, mouth, pharynx and other cavity regions as the cancer sites, the estimated total cases at the sites will be around 116,900 in both sexes in which 83,020 new cancer cases are estimated in males and 33,880 new cases are estimated in females and the estimated deaths are around 24,460 in US [4]. The incidence of HNC is increasing and is expected to rise to 1.08 million new cases per year by 2030 [2].

Longstanding evidence suggests that high use of tobacco is linked to the development of HNSCC. Tobacco usage and smoking remain a major risk factor for HNC occurrence and progression. Chewing areca nuts, with or without tobacco, is also considered a predominant risk factor [1,5], and Chronic alcohol consumption is another major global determinant. However, the combination of tobacco usage along with alcohol consumption increased the risk to approximately 72% in HNSCC patients [1]. Given diverse etiologies, metabolomic profiling offers a unique way to capture both viral- and chemical-carcinogen-driven metabolite shifts. Recently, human papillomavirus (HPV) subtypes 16 and 18, which are considered high oncogenic risk factors, have been associated with the increased risk of Oropharyngeal cancer, especially in Western countries [6]. Overall survival (OS) rate in HNSCC cancers varies significantly with correlation to HPV status. HPV-associated oropharyngeal cancers have a survival rate of approximately 80% and have a higher survival rate compared to stage III-IV patients with locoregionally advanced laryngeal carcinoma [7,8]. HPV viral infections and HPV-positive cancers can be prevented by treating with commercialized HPV vaccines; hence, it is considered feasible to control the spread of HPV positive HNSCC globally by mass vaccination [2]. Epstein–Barr virus infection is another risk factor contributing to nasopharyngeal carcinoma (NPC) [9].

Considering the percentage of HNSCC as a high-incidence cancer globally, there is a critical need for early diagnosis to improve patient outcomes. Metabolomics offers a promising approach for discovering biomarkers and metabolic pathways that are crucial for assessing HNSCC risk and detecting it early [10,11]. Metabolomics has become an invaluable tool in the quest for new cancer biomarkers. However, the complexity of metabolomic data poses challenges, necessitating advanced techniques such as deep learning and data mining for interpretation [12,13]. Furthermore, metabolite concentrations vary widely, ranging from nanomolar to millimolar, and can be influenced by factors such as diet, age, and medication. Despite these hurdles, studies have identified potential metabolomic biomarkers in HNSCC [14]. Nevertheless, extensive validation studies are crucial to confirm the diagnostic utility of these metabolites in head and neck neoplasms. Presently, research on metabolomic biomarkers for cancer diagnosis remains in the exploratory stage [15,16].

Several recent reviews have addressed metabolic reprogramming in HNSCC, immunometabolism, or mass spectrometry-based metabolomics as independent topics. In contrast, the present review uniquely integrates these domains into a unified immune-metabolomic framework, emphasizing how tumor intrinsic metabolic rewiring intersects with immune dysfunction, stromal remodeling, and therapeutic resistance in HNSCC. Importantly, we move beyond descriptive cataloguing by critically reconciling conflicting metabolomic findings across HPV status, anatomical subsites, disease stages, and analytical platforms. We further highlight emerging advances in spatial and single-cell metabolomics, which are reshaping our understanding of intratumoral metabolic heterogeneity and enabling context-aware biomarker discovery. By bridging mechanistic metabolism with clinical translation, this review provides a roadmap for leveraging metabolomics toward precision oncology in HNSCC.

### Literature Search and Study Selection

This article is a narrative, integrative review designed to synthesize current knowledge on metabolic reprogramming and metabolomics in HNC, with an emphasis on biological context, reproducibility, and translational relevance rather than exhaustive quantitative aggregation.

A structured literature search was conducted using PubMed, Scopus, and Web of Science databases. Articles published between 2005 and 2025 were considered to capture the emergence and maturation of metabolomics technologies applied to HNC. Search terms included combinations of: “head and neck cancer,” “HNSCC,” “oral squamous cell carcinoma,” “metabolomics,” “metabolic reprogramming,” “immunometabolism,” “tumor microenvironment,” “LC-MS,” “GC-MS,” “NMR,” “saliva,” “plasma,” “urine,” and “tissue metabolomics”. Reference lists of relevant reviews and primary studies were also manually screened to identify additional key publications.

Studies included if they: (i) involved human HNC patient samples, tumor tissues, or validated HNC cell line models; (ii) employed metabolomics or metabolic profiling approaches (MS- or NMR-based); and (iii) reported differential metabolites, pathways, or clinically relevant metabolic associations.

Non-English articles, case reports, conference abstracts, purely in silico studies, and studies lacking appropriate control groups were excluded.

Given the heterogeneity of metabolomics platforms and study designs, articles were prioritized based on reproducibility, cohort size, biological relevance, and clinical interpretability, rather than solely on statistical significance. Greater emphasis was placed on metabolites and pathways reported across multiple independent cohorts, biological matrices, or analytical platforms. Conflicting findings were retained and critically discussed to highlight metabolic plasticity, methodological limitations, and unresolved questions in the field.

## 2. Metabolic Reprogramming in HNC: Core Hallmarks

### 2.1. The Warburg Effect and Aerobic Glycolysis

By regulating energy metabolism, tumor cell reprogramming accelerates cell growth, proliferation, and differentiation. Additionally, it is considered a distinct hallmark of tumor cells, characterized by glutaminolysis, aerobic glycolysis, and increased lipid synthesis [17]. Normal differentiated cells primarily rely on mitochondrial oxidative phosphorylation to generate the energy required for cellular processes. However, most cancer cells instead rely on aerobic glycolysis, a phenomenon termed the “Warburg effect,” discovered by Otto Heinrich Warburg in 1920 [18]. Although aerobic glycolysis is widely regarded as a hallmark of cancer metabolism, evidence for a uniform Warburg phenotype in Oral Squamous Cell Carcinoma (OSCC) remains inconsistent. Several tissue-based NMR studies report unchanged lactate levels in OSCC compared with adjacent normal tissue, suggesting preserved mitochondrial oxidative metabolism in subsets of tumors. In contrast, CE-TOF-MS and HR-MAS-NMR studies consistently demonstrate elevated lactate accumulation and depletion of upstream glycolytic intermediates, indicative of enhanced glycolytic flux [19,20,21].

These discrepancies likely reflect metabolic plasticity rather than experimental contradiction. OSCC tumors exhibit dynamic switching between glycolysis and oxidative phosphorylation depending on oxygen availability, stromal interactions, and oncogenic drivers [22,23]

Furthermore, analytical platform sensitivity, tissue handling, and normalization strategies strongly influence lactate detection. Importantly, lactate levels alone may not accurately reflect glycolytic flux, as lactate export, stromal uptake, and immune cell consumption further modulate steady-state concentrations in the tumor microenvironment (TME). All proliferating cells, including cancer cells, adapt to facilitate the incorporation of nutrients into biomass (i.e., nucleotides, amino acids, and lipids) by activating several signaling mechanisms, as explained in detail in later sections.

### 2.2. Glutaminolysis and Amino Acid Metabolism

**Figure 1:**
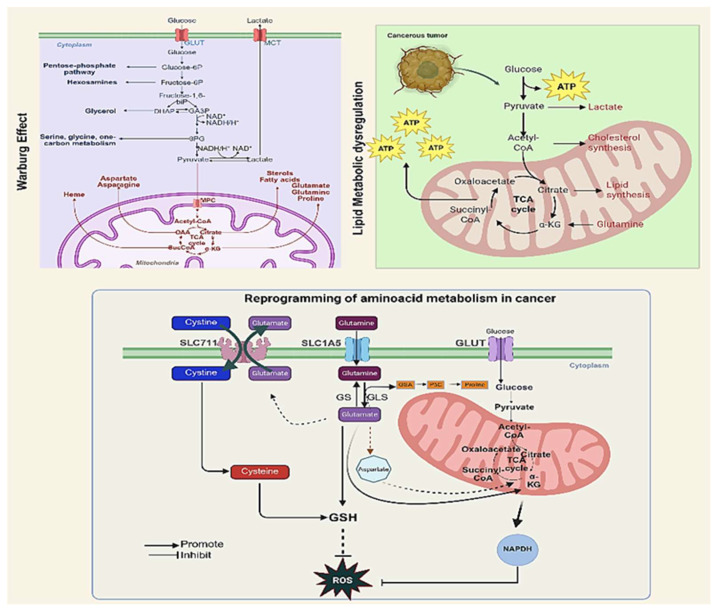
Core hallmarks of Metabolic reprogramming—Warburg effect, Lipid metabolic dysregulation, and reprogramming of amino acid metabolism in cancer.

Beyond glucose, many HNCs are “glutamine-addicted”, heavily relying on glutaminolysis to fuel the tricarboxylic acid cycle and biosynthesis. Glutamine is the most abundant amino acid in blood and a versatile nutrient that cancer cells use for energy, macromolecular synthesis, and maintenance of redox balance. While earlier studies suggested HNC is primarily dependent on glucose for energy, it is now clear that glutamine is also critical for HNSCC tumorigenesis [24]. HNSCC tumors frequently overexpress glutamine transporters such as SLC1A5/ASCT2 (and the neutral amino acid exchanger LAT1) as well as glutaminase (GLS), the enzyme converting glutamine to glutamate [24]. High expression of ASCT2 and GLS correlates with poor survival in HNSCC, reflecting the importance of glutamine-fueled metabolism. Functionally, silencing or inhibiting ASCT2 in HNSCC cell lines results in marked suppression of glutamine uptake and its downstream metabolic products, leading to decreased intracellular glutathione and increased oxidative stress, which impairs growth and enhances apoptosis [24]. Glutamine also activates mTORC1 and other growth signaling pathways; consistently, glutamine-starved HNSCC cells show mTORC1 suppression and even induction of autophagy [25]. Therefore, glutamine reprogramming in HNSCC supports both anabolism and survival under oxidative and nutrient stress. Therapeutically, targeting this vulnerability has shown promise by blocking ASCT2 or GLS, resulting in reduced HNSCC xenograft growth and synergizing with targeted therapy when combined with the EGFR inhibitor cetuximab [24]. Current ongoing research includes examining GLS inhibitors (teleglenastat/CB-839) in solid tumors, including HNSCC, and early results support that glutamine-addicted HNCs may respond favorably to such strategies when combined with standard treatments [26]. Glutamine thus serves as a key anaplerotic substrate and a precursor for glutathione (GSH), making it indispensable for redox homeostasis in HNSCC cells (Figure 1).

### 2.3. Lipid Metabolism Dysregulation

Alterations in lipid metabolism are increasingly recognized in HNSCCs, reflecting the tumor’s need for structural components (membranes), energy storage/utilization, and lipid-derived signaling molecules. HNSCC tumors exhibit numerous abnormalities in lipid profiles and enzyme regulation. For example, comparative lipidomic analyses have shown accumulation of specific fatty acids, including palmitic acid (C16:0), in HNSCC tissues, alongside reductions in others, such as ceramides. At the molecular level, there is overexpression of key lipogenic enzymes, with some studies consistently reporting upregulation of fatty acid synthase (FASN) and acetyl-CoA carboxylase (ACC) in HNSCC [25], driven in part by oncogenic signaling, in which PI3K/AKT/mTOR upregulates SREBP1, a master lipogenic transcription factor. These enzymes enable the de novo synthesis of fatty acids, supplying rapidly proliferating cancer cells with phospholipids and triglycerides. High FASN activity in HNSCC has correlated with the worst prognosis and is being explored as a therapeutic target. Concurrently, HNSCC cells often increase exogenous lipid uptake and lipid trafficking. The fatty acid importer CD36 is highly expressed in many HNCs, and its upregulation correlates with advanced tumors and metastasis [25] (Figure 1).

## 3. Key Oncogenic Pathways Driving Metabolic Changes

Oncogenic mutations within the cell result in increased nutrient uptake, particularly glucose, to meet the bioenergetic demands of cell growth and proliferation.

### 3.1. PI3K/AKT/mTOR Signaling Axis

In normal physiology, the PI3K/AKT/mTOR axis regulates cell survival, growth, and metabolism. Alterations result in the malignant phenotype that characterizes HNSCC and other cancers. A detailed review of the role of the PI3K/AKT/mTOR axis in HNSCC has been discussed elsewhere [27].

### 3.2. Hypoxia-Inducible Factors (HIFs)

Hypoxia-inducible factors (HIFs) are heterodimeric transcription factors composed of an oxygen-sensitive α subunit and a constitutive β subunit. In oxygen-rich (normoxic) conditions, HIF-α proteins are continuously synthesized but are rapidly marked for destruction by oxygen-dependent enzymes. Prolyl hydroxylase domain proteins (PHDs) use oxygen and α-ketoglutarate as substrates to hydroxylate specific proline residues on HIF-α. This modification creates a binding site for the von Hippel-Lindau (VHL) protein, the recognition subunit of an E3 ubiquitin ligase. VHL binding leads to ubiquitination and proteasomal degradation of HIF-α, thus keeping HIF activity low under normoxia [28]. When oxygen levels decrease, PHD enzymes become inhibited, preventing VHL-mediated degradation of HIF-α. This further results in the stabilization of HIF-α accumulation in the nucleus, dimerizes with HIF-1β, and binds to hypoxia-responsive elements (HREs, with core sequence 5′-RTGTG-3′) in target genes to activate their transcription [29]. The oxygen-sensing mechanism enables cells to acutely respond to hypoxia by activating a broad transcriptional program.

The metabolic functions of HIF-1α and HIF-2α are co-opted to support malignant growth. As discussed previously, many of the HIF-driven changes, including glycolysis, glutamine dependence, lipogenesis, and ROS management, are hallmarks of cancer metabolism. HNCs, including HNSCCs, illustrate well the impact of hypoxia-inducible pathways on tumor metabolism and behavior. These cancers often contain large hypoxic regions due to an inadequate and disorganized tumor blood supply [30,31]. Clinically, markers of hypoxia (such as HIF-1α protein levels or hypoxia gene signatures) have been associated with more aggressive disease and worse outcomes in HNC patients [28,30]. For example, elevated HIF-1α in HNSCC correlates with increased risk of treatment failure and reduced overall survival, making HIF-1α a known indicator of poor prognosis in this disease [28].

HIF-driven metabolism is tightly intertwined with oncogenic pathways. The PI3K/AKT/mTOR pathway, for instance, directly stimulates glucose uptake and glycolysis, and HIF-1α protein synthesis [32]. One direct consequence of HIF activation in head and neck tumors is a shift to high-rate glycolysis. FDG-PET scans are often positive in HNSCC, reflecting the Warburg phenotype. At the molecular level, HIF-1α upregulates glucose transporters and glycolytic enzymes in these cancers, as in other tumors. GLUT1 is commonly overexpressed in HNSCC and is linked to tumor hypoxia; importantly, high GLUT expression in HNSCC has been found to correlate with worse patient outcomes, partly because it enables greater glucose consumption and reduces apoptosis [30]. A study by Grimm et al. showed that patients with high GLUT1 expression in their tumors had a significantly poorer prognosis than those with low GLUT1 expression [33]. Likewise, lactate dehydrogenase-A is frequently upregulated in HNCs in a HIF-1-dependent manner. High LDH-5 expression in tumor tissue or elevated LDH, with statistically significant p values (<0.05) in serum, has been linked to metastasis and reduced survival in HNSCC. These clinical correlations underscore that the HIF-driven glycolytic switch in HNSCC portends more aggressive cancer behavior [34].

### 3.3. MYC, TP53, and Other Metabolic Regulators

C-Myc acts as a central oncogenic transcription factor, frequently activated in HNSCC either by amplification or by HPV E7-induced bypass of Rb. The C-Myc gene upregulates nearly all facets of anabolic metabolism, including glycolytic enzymes, glutamine transport and catabolism, serine/glycine synthesis by regulating ATF4, and nucleotide biosynthesis. C-Myc also represses microRNAs that normally dampen the expression of metabolic genes. Currently, therapeutic small-molecular targets for C-Myc are in early development. A recent approach in HNSCC combined a GLS1 inhibitor with a prototype c-Myc inhibitor, yielding synergistic suppression of tumor growth [35]. This approach suggested that dual targeting of C-Myc and metabolic pathways could represent a potential therapeutic strategy, essentially starving the cancer cell by blocking the metabolic master switch.

The tumor suppressor p53 is mutated in 60–80% of HPV-negative HNSCC and is functionally inactivated in HPV-positive cases by viral E6. Wild-type p53 normally acts as a metabolic checkpoint by inducing TIGAR (TP53-inducible glycolysis and apoptosis regulator) and can also induce GLS2, a form of glutaminase that feeds the TCA cycle and enhances mitochondrial respiration and antioxidant capacity [36]. P53 also upregulates cytochrome c oxidase assembly factor (SCO2) and promotes OXPHOS, resulting in downregulation of the GLUT1 transporter (SLC2A1) and cystine transporter (SLC7A11) to limit glycolysis and antioxidant import [37].

Metabolic reprogramming in HNSCC is further shaped by HPV status, which imposes distinct oncogenic and metabolic constraints. HPV-positive tumors, characterized by viral E6/E7-mediated inactivation of p53 and Rb, display enhanced glycolysis coupled with reduced oxidative phosphorylation capacity, consistent with a reliance on anabolic glucose metabolism [38,39].

In contrast, HPV-negative tumors frequently harboring TP53 mutations retain greater mitochondrial respiratory flexibility and exhibit increased glutaminolysis and fatty acid oxidation. These metabolic distinctions may partly explain the superior treatment responsiveness of HPV-positive HNSCC, as glycolysis-dependent tumors appear more sensitive to radiotherapy and metabolic stress [40]. However, the extent to which HPV-specific metabolic vulnerabilities can be therapeutically exploited remains an open question requiring prospective validation.

## 4. Tumor Microenvironment and Immune Metabolism in HNC

### 4.1. Hypoxia and Acidic Microenvironment

Tumor cells often reside in a microenvironment with inadequate blood supply, leading to intratumoral hypoxia. It is estimated that more than half of the solid tumors contain hypoxic regions. HIF-1α is frequently stabilized in these regions and serves as a key regulator of tumor metabolism and progression [30]. Clinically, high HIF-1α levels in tumors have been correlated with poor patient outcomes in many types of cancer, including brain, breast, colon, lung, pancreas, and head and neck cancers [28]. This is because HIF activation not only enables cancer cells to survive under low-oxygen conditions but also drives aggressive traits, including angiogenesis, invasion, metastasis, and therapy resistance. Furthermore, acidic extracellular pH impairs immune cell function and contributes to the immunosuppressive TME by limiting cytotoxic T lymphocyte activity and promoting regulatory T cell (Treg) differentiation [41,42].

### 4.2. Crosstalk with Cancer-Associated Fibroblasts and Immune Cells

Cancer-associated fibroblasts (CAFs), prominent stromal cells in the HNC TME, actively communicate with tumor and immune cells, significantly influencing tumor progression and immune responses. CAFs secrete cytokines, growth factors, and extracellular matrix proteins that enhance tumor growth, invasion, and angiogenesis [43]. The interplay between CAFs and tumor cells further exacerbates metabolic alterations, promoting glycolysis and lactate secretion, thus sustaining the acidic and immunosuppressive conditions within tumors [44]. CAFs can also modulate immune cell filtration and function by secreting immunosuppressive factors such as transforming growth factor-beta (TGF-β), interleukin-6 (IL-6), and chemokine ligands (CCL2, CCL5), which facilitate the recruitment and activation of immunosuppressive cells, including myeloid-derived suppressor cells (MDSCs), tumor-associated macrophages (TAMs), and Tregs [45,46].

### 4.3. Immunometabolism and Immune Evasion

**Figure 2:**
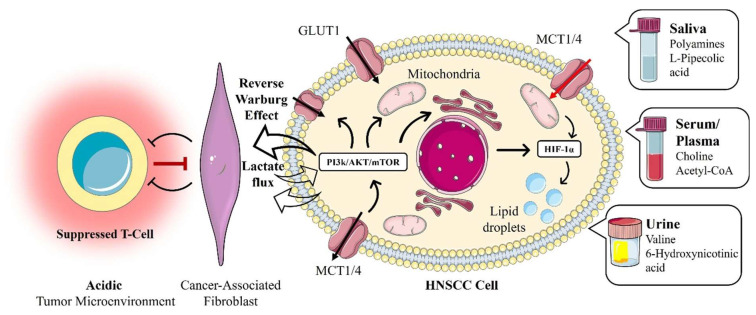
Integrative Metabolic Landscape of HNSCC. This schematic bridges intracellular metabolic reprogramming, tumor-stromal crosstalk, and systemic biomarker detection. Oncogenic drivers (PI3K/AKT/mTOR, HIF-α) fuel the Warburg effect, glutamine addiction, and lipid dysregulation. These pathways shape an acidic, immunosuppressive TME while simultaneously shedding distinct metabolic signatures into saliva, blood, and urine, offering non-invasive avenues for early detection, prognostic monitoring, and therapeutic stratification.

Hypoxia-inducible pathways also facilitate immune evasion and therapy resistance in tumors via metabolic means. Tumor cells and CAFs create a nutrient-depleted, hypoxic, and acidic microenvironment that profoundly reshapes immune cell metabolism, impairing effective antitumor immunity. Effector T cells, for example, rely heavily on glycolysis for activation and effector functions; thus, glucose deprivation resulting from high tumor cell glycolysis suppresses their activity and proliferation [45]. Additionally, elevated lactate levels in the TME suppress the function of cytotoxic T lymphocytes (CTLs) and natural killer (NK) cells, further facilitating immune escape [47]. Lactate and acidosis (products of HIF-driven metabolism) can suppress antitumor immune effectors and promote a suppressive tumor microenvironment. HIF-1 also induces immune checkpoint molecules, such as PD-L1, in hypoxic cancer cells, linking metabolism to immune escape. Meanwhile, as noted, antioxidant adaptations confer resistance to radiation and to some chemotherapies that rely on ROS to kill tumor cells [4]. Additionally, HIF-induced angiogenesis (via VEGF and other factors) can transiently improve perfusion but often results in disorganized vasculature, enabling further tumor expansion. These complex immune-metabolic interactions highlight novel opportunities for targeted therapeutic interventions to restore immune function in HNC. The convergence of these intracellular metabolic shifts (such as the Warburg effect and lipid dysregulation), their profound impact on the tumor microenvironment, and their subsequent manifestation as detectable biomarkers in clinical biofluids are comprehensively mapped in Figure 2.

## 5. Metabolomics in HNC: Techniques and Applications

**Figure 3:**
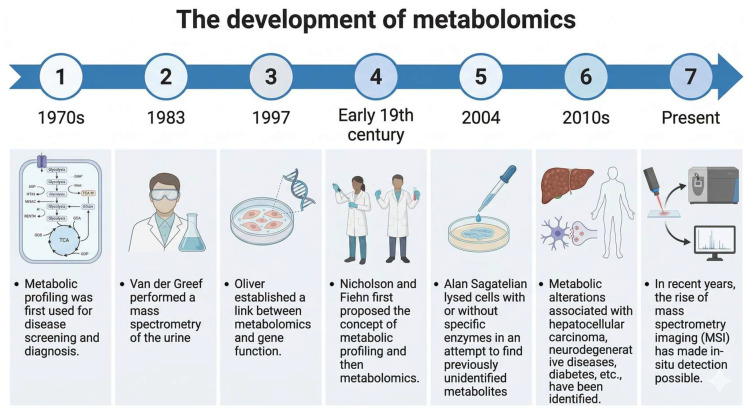
Evolution of metabolomics strategies in HNSCC research. This schematic depicts the methodological progression of metabolomics applications in HNSCC, beginning with early bulk profiling platforms such as NMR and GC–MS, advancing to high-resolution LC–MS, and extending to spatially resolved mass spectrometry imaging and emerging single-cell–informed techniques. The timeline reflects general trends in the published literature rather than a formal bibliometric assessment.

Metabolomics, a comprehensive analysis of low-molecular-weight metabolites within biological systems, has significantly advanced cancer research. By capturing the downstream effects of gene expression and environmental influences, metabolomics provides real-time functional insights into tumor biology and offers substantial potential for biomarker discovery, therapeutic target identification, and precision diagnostics in HNSCC [2,3] (Figure 3).

Among the available analytical platforms, liquid chromatography-mass spectrometry (LC-MS) is widely used for its high sensitivity, broad metabolite coverage, and versatility across both targeted and untargeted metabolomic workflows. In HNSCC studies, LC-MS-based metabolomics has revealed several dysregulated metabolic pathways, including glycolysis, amino acid metabolism, and lipid metabolism, enabling discrimination between tumor tissues and normal epithelial counterparts [2,4]. The integration of tandem Mass Spectrometry (MS/MS) further enhances identification by improving analytical specificity and structural confirmation.

The inherent chemical and physical diversity of metabolites presents significant analytical challenges in metabolomic profiling. To address this, researchers often integrate complementary techniques, such as nuclear magnetic resonance spectroscopy (NMR) and MS, with chromatographic separation strategies to improve metabolite detection and resolution in complex biological samples. Future advancements in metabolomics are expected to focus on developing sophisticated statistical algorithms and integrating large-scale metabolite databases, which are essential for deciphering complex metabolic networks.

### 5.1. Metabolomics Approaches: Targeted and Untargeted Strategies

Metabolomic investigations in HNSCC typically employ two complementary analytical strategies: targeted and untargeted metabolomics. Targeted metabolomics focuses on the accurate quantification of pre-defined metabolites using validated standards, enabling precise measurement of specific metabolic pathways implicated in tumor progression or treatment response [48]. In contrast, untargeted metabolomics provides a comprehensive, hypothesis-generating survey of metabolic alterations, facilitating the discovery of novel biomarkers associated with HNSCC progression and development. [49].

Untargeted approaches are frequently used during early-stage biomarker discovery to identify dysregulated metabolic pathways in tumor tissues and biofluids [50]. Studies using untargeted metabolomics have reported significant alterations in glycolysis, amino acid metabolism, and lipid metabolism in HNSCC, reflecting metabolomic reprogramming associated with tumor growth and invasion [2,4,51,52]. However, untargeted workflows generate large datasets that require further validation due to potential false positives and analytical variability. Consequently, targeted metabolomic assays are typically used in subsequent validation phases to confirm candidate biomarkers and enable reproducible quantitative measurements [49].

The combined use of discovery-based untargeted profiling followed by targeted validation has become a standard strategy for translating metabolomic findings into clinically relevant biomarkers for HNSCC [52,53].

### 5.2. Sample Collection and Processing in HNSCC Metabolomics

The reliability of metabolomic analyses is strongly influenced by sample collection, handling, and storage conditions. In HNSCC metabolomic studies, commonly analysed biological matrices include tumor tissues, plasma, serum, urine, and saliva [54]. Among these, saliva has emerged as a particularly promising biofluid for metabolomic profiling due to its non-invasive collection and its direct interaction with oral tumors [52].

Several studies have identified salivary metabolic signatures that distinguish HNSCC patients from healthy individuals, highlighting the potential of metabolomics for early disease detection and monitoring. Similarly, plasma and serum metabolomics have revealed systemic metabolic alterations associated with tumor metabolism and treatment response [52,53,54,55].

However, metabolomic measurements are highly sensitive to preclinical factors such as diet, medication, circadian variation, and sample processing protocols [56]. Variability in extraction procedures, preservation methods, and storage conditions can significantly influence metabolic stability and detection. These methodological differences have contributed to inconsistencies between metabolomics studies and remain a major challenge for biomarker reproducibility in HNSCC research. Standard sample preparation protocols and appropriate quality control procedures are therefore essential to improve data compatibility across studies [57,58].

### 5.3. Analytical Platforms in HNSCC Metabolomics

Two principal analytical platforms dominate metabolomics research: nuclear magnetic resonance (NMR) spectroscopy and mass spectrometry (MS). Each technique offers distinct advantages for investigating metabolic alterations associated with HNSCC.

NMR spectroscopy provides highly reproducible and quantitative metabolite measurements with minimal sample preparation, making it particularly suitable for large-scale biofluid studies. NMR-based metabolomics has been applied to identify metabolic signatures associated with HNSCC progression, treatment response, and tumor metabolism [51,59,60]. However, NMR’s relatively low sensitivity limits its ability to detect metabolites at very low concentrations [61,62].

Mass-spectrometry-based metabolomics has therefore become the most widely used approach in HNSCC research due to its high sensitivity and broad metabolite coverage. MS platforms are typically coupled with chromatographic separation techniques such as LC-MS or GC-MS, enabling the detection of hundreds to thousands of metabolites within complex biological samples. LC-MS is particularly useful for profiling polar metabolites, lipids, and signaling molecules in tumor tissues and plasma, whereas GC-MS is commonly used to analyze volatile metabolites and small organic compounds associated with oral cancers [63,64,65].

Despite these advantages, variability across analytical platforms remains a major challenge in metabolomics research. Differences in instrumentation, ionization methods, sample preparation protocols, and data processing pipelines can lead to inconsistencies between studies and limit the reproducibility of candidate biomarkers. In HNSCC metabolomics, the lack of standardized analytical workflows and validation across independent patient cohorts has been identified as a key barrier to clinical translation. Addressing these challenges will require improved methodological standardization, larger multicenter studies, and the integration of metabolomic data with genomic and proteomic datasets to better understand metabolic heterogeneity in HNSCC.

Table 1 provides an overview of the application of these MS-based techniques.

### 5.4. Biological Samples Used for Head and Neck Cancer Metabolomics

#### 5.4.1. Saliva and Plasma as Biofluids for Non-Invasive Diagnostics

Saliva is a complex biological fluid containing a wide array of constituents such as proteins, electrolytes, lipids, trace metals, and various metabolites. It performs essential physiological functions, including facilitating speech, taste, digestion, and tissue lubrication; promoting tooth remineralization and detoxification; and exhibiting antimicrobial and antiviral properties [71,72]. Owing to its non-invasive and easily accessible nature, saliva has become an attractive biofluid for omics-based research [73,74]. Metabolomics investigations commonly use either stimulated or unstimulated whole saliva, and because of their differing biochemical compositions, it is crucial that studies clearly specify the saliva type used [74,75,76].

Metabolites in saliva originate from multiple sources, including salivary glands, systemic circulation, and cells within the oral cavity, rendering it a reflective medium for both local and physiological changes [77]. This characteristic underpins its emerging utility for early detection and monitoring of diseases, including HNCs (particularly OSCC), which constitute the majority of HNC cases and are characterized by a poor prognosis and high mortality rates.

Recent studies have explored salivary metabolites to distinguish OSCC from precancerous lesions such as oral lichen planus (OLP) and oral leukoplakia (OLK). For instance, Yan et al. applied hierarchical principal component analysis (PCA) and discriminant analysis to successfully differentiate OSCC from OLP and OLK, although differentiation from OLK and OLP was less distinct [78]. In a separate study, Almadori et al. reported significantly elevated salivary glutathione levels in patients with oral and pharyngeal squamous cell carcinoma compared to healthy individuals, suggesting glutathione as a potential epidemiological biomarker for OSCC risk assessment [79].

Proteomic analyses have also identified promising salivary biomarkers. Using two-dimensional gel electrophoresis combined with MALDI-TOF-MS, researchers observed increased transferrin expression in OSCC patients. Western blot and immunohistochemical assays further confirmed these findings, and a positive correlation between transferrin levels and tumor size and stage was observed, indicating its potential utility for early diagnosis [80].

Polyamines are organic cations that are essential for cellular proliferation and differentiation. They frequently elevate in cancerous tissues and are linked to increased tumor invasiveness and metastasis [81,82,83]. Sugimoto et al. employed CE-TOF-MS and reported higher polyamine levels in OSCC compared to breast and pancreatic cancers, as well as to healthy controls [52]. This highlights the discriminatory power of polyamines as cancer-specific biomarkers.

Using ultra-performance liquid chromatography coupled with quadrupole time-of-flight mass spectrometry (UPLC-Q-TOF-MS), Wei et al. identified a panel of five salivary metabolites-γ-amino butyric acid, phenylalanine, valine, n-eicosanoic acid, and lactic acid-that differentiated OSCC from healthy samples, offering promise for clinical application in OSCC detection [81,84]. Similarly, Ishikawa and colleagues used CE-TOF-MS to analyze saliva and tumor tissues from OSCC patients, identifying 17 metabolites with consistent alterations across both sample types. Among them, SAM and pipecolate were significantly elevated in both tissue and saliva, supporting their potential role as dual markers for OSCC [51].

Further supporting these findings, a study involving a Japanese population identified 25 salivary metabolites capable of distinguishing OSCC patients from healthy individuals, with seven metabolites—taurine, valine, leucine, isoleucine, choline, cadaverine, and tryptophan—overlapping with previous discoveries. More recently, dual-platform metabolomics using LC/LC-MS and GC-MS was applied to assess oral rinses and tissue samples from HNSCC patients [85]. The analysis revealed 23 metabolites in oral rinses from cancer patients, compared with 12 in healthy controls, with β-alanine, hydroxyisovalerate, tryptophan, and hexanoylcarnitine significantly elevated in cancer cases (Table 2). Additionally, tumor tissues exhibited elevated levels of key metabolic intermediates, including 2-hydroxyglutarate and glycerol-3-monophosphate-metabolites linked to TCA cycle disruptions.

Collectively, these studies highlight the strong diagnostic potential of salivary metabolomics for early detection and prognosis of HNCs, positioning saliva as a valuable, non-invasive medium for biomarker discovery.

Plasma offers a window into systemic metabolomic alterations associated with cancer progression. It is widely used in clinical metabolomics due to its stability and rich metabolite content. The role of Plasma metabolomics in HNC has been highlighted in the earlier sections of this article. Furthermore, it enables longitudinal monitoring of treatment response and detection [86].

**Table 2:** Summary of metabolites identified in different biological matrices of head and neck cancer (HNC) subjects.

Biological Matrix	Cancer Type	Study Cohort (n)	Detection Method	Key Dysregulated Metabolites/Pathways	Reproducibility	Clinical Relevance/Interpretation	Reference
Saliva	HNC	10 HNC, 9 pSS, 10 HC	UPLC-Orbitrap-MS	Pyrimidine nucleotide and amino acid metabolism	Moderate	Reflects inflammatory–neoplastic metabolic overlap	[87]
	HNC	35 HNC, 72 HC	LC-MS/MS	↑ N1-acetylspermine (polyamine metabolism)	High	Non-invasive diagnostic biomarker	[88]
	SCCT	20 SCCT, 10 HC	LC-MS/MS	N-acetyl-D-glucosamine, pipecolic acid, carnitine (ROC AUC 0.901)	Moderate	Multi-metabolite diagnostic panel	[89]
	HNSCC	8 HNSCC, 30 HC	^1^H NMR	↑ fucose, 1,2-propanediol; ↓ proline	Low-moderate	Metabolic fingerprinting	[90]
	HNSCC	50 HNSCC, 77 HC	HPLC	↑ glutathione (redox metabolism)	Moderate	Oxidative stress adaptation	[73]
	OSCC/OLP/OLK	20 OSCC, 20 OLP, 70 OLK, 11 HC	HPLC/CE-TOF-MS	Distinct metabolic profiles across lesion stages	Moderate	Premalignant vs. malignant stratification	[72]
	OSCC/OLK	37 OSCC, 32 OLK, 34 HC	UPLC-Q-TOF-MS	Amino acids, energy metabolites	Moderate	Lesion discrimination	[78]
	OSCC	41 OSCC, 30 HC	MALDI-TOF-MS	↑ transferrin	Low	Limited specificity	[74]
	OSCC	69 OSCC, 87 HC	CE-TOF-MS	Energy and amino acid metabolism	Moderate	Outcome prediction	[52]
	OSCC	44 OSCC, 20 HC	CE-TOF-MS	↑ lactate, arginine, ornithine; ↓ glycolytic intermediates	High	Glycolytic rewiring; hypoxia	[51]
	OSCC	22 OSCC, 21 HC	CE-MS	Differential amino acids and energy metabolites	Moderate	Diagnostic discrimination	[85]
Urine	HNC	39 HNC, 89 HC	LC-MS/MS	↑ acetylated polyamines	High	Systemic tumor burden; monitoring	[88]
	OSCC/OLK	37 OSCC, 32 OLK, 34 HC	GC-MS	↑ valine, alanine; ↓ Hippurate	Moderate–High	High-accuracy diagnostic model	[91]
	Laryngeal cancer	37 cancer, 29 HC	LC-QTOF-MS	Fatty acids, sphingolipid metabolism	Moderate	Lipid remodeling	[92]
Blood	HNSCC	137 HNSCC	LC-HRMS	Glycolysis and OXPHOS metabolites	High	Prognostic; independent of HPV/smoking	[93]
	HNPGL	59 patients, 24 HC	FIA-MS/MS	TCA rewiring, glutaminolysis	Moderate	Metabolic vulnerability	[94]
	HNC (RT response)	20 HCN	GC-MS	↑ 3-hydroxybutyrate	Low-Moderate	Therapy response marker	[95]
	HNSCC	25 patients	GC-MS	↓ amino acids; ↑ glycolysis	Moderate	Systemic metabolic stress	[96]
	OSCC/OLK	100 OSCC, 100 OLK, 75 HC	^1^H NMR	↑ choline, acetate; ↓ glutamine	High	Progression marker	[97]
	OSCC/OLK	33 OSCC, 5 OLK, 28 HC	^1^H NMR	Altered amino acid and energy metabolism	Moderate	Diagnostic discrimination	[98]
	OSCC	15 OSCC, 10 HC	^1^H/^2^D NMR	Ketone bodies, TCA intermediates	Moderate	Energy reprogramming	[99]
	Laryngeal cancer	39 cancer, 53 HC	QTrap-MS	Arginine, ornithine, acylcarnitines	Moderate	Metabolic fingerprinting	[100]
Tissue	HNSCC	7 tumor/adjacent	LC-MS, FT-ICR, GC-MS	↑ 2-hydroxyglutarate; acylcarnitines	Moderate	Oncometabolite signaling	[101]
	Salivary gland cancer	11 tumor/adjacent	MALDI-MSI	↑ glycerophospholipids; ↓ sphingomyelins	Moderate	Spatial lipid remodeling	[102]
	HNSCC	25 paired samples	GC-MS	↑ amino acids; ↓ glycolysis	Moderate	Metabolic heterogeneity	[103]
	OSCC/OSF	21 OSCC, 15 OSF, 15 HC	GC-MS	↓ multiple amino acids	Moderate	Disease progression	[104]
	HNSCC	85 HNSCC, 50 HC	^1^H MRS	↑ choline, lactate, taurine	High	Hallmark tumor metabolism	[105]
	OSCC	159 paired tissues	HR-MAS NMR	↑ lactate, choline, amino acids	High	Robust tissue signature	[106]
	HNSCC + LN-Met	22 matched samples	HR-MAS ^1^H NMR	↑ lactate, amino acids; ↓ triglycerides	High	Metastatic progression	[84]
	OSCC	18 OSCC, 12 HC	^1^H/^13^C NMR	Choline breakdown, malonate	Moderate	Membrane metabolism	[107]
	OSCC/OSF	15 per group	Nano-LC-MALDI-MS	Lipid remodeling	Moderate	Aggressiveness	[108]
Cell lines	HNSCC	HPV^+^/HPV^−^ lines	CE-FTMS	HPV-dependent glycolysis–OXPHOS balance	Moderate	Mechanistic insight	[40]
	HNSCC	5 HNSCC, 3 NHOK	^1^H NMR	Choline phospholipid metabolism	Moderate	Target discovery	[109]
	OSCC	SCC15, HSC-3	FTIR imaging	Lipid and membrane alterations	Low	Phenotypic support	[103]
	HNSCC	CSC vs. non-CSC	CapIC-MS	Glycolysis and TCA changes	Moderate	Stemness metabolism	[110]

#### 5.4.2. Blood and Urine Metabolomics

Researchers have extensively explored metabolites in blood and urine to assess their biological significance, prevalence, and diagnostic potential in various diseases, including cancer [111,112]. Among biofluids, blood, particularly plasma and serum, has been the predominant focus of metabolomic research due to its rich and diverse metabolite composition, comprising hormones, electrolytes, metabolic byproducts, nutrients, and organic waste products [112,113]. The aqueous phases of serum and plasma share similar metabolite profiles, enabling parallel analysis [111]. Various pathologies, including cancer, alter the chemical and metabolic profiles of blood, underscoring its potential as a diagnostic tool [112,114]. Multiple studies have reported distinctive metabolic signatures in serum and plasma from patients with HNC. Choline consistently emerged as a significantly elevated metabolite across various HNC sample analyses. Specifically, studies of OSCC have repeatedly demonstrated elevated levels of choline-containing metabolites compared with healthy controls [78,115]. Choline, an essential nutrient, undergoes phosphorylation by choline kinase to produce phosphocholine, a precursor for phosphatidylcholine synthesis, which is integral to cell membrane formation [116].

Elevated phosphocholine levels are indicative of enhanced cellular proliferation, viability, and metastatic potential, rendering choline metabolism a critical biomarker and therapeutic target in cancer biology [117,118]. Indeed, extensive research has linked choline metabolism intricately with oncogenic signaling pathways, reinforcing its role in tumorigenesis and metastasis [118,119,120].

Distinct metabolic alterations in serum and plasma have also been documented in HNC patients. Tiziani et al. utilized NMR spectroscopy and identified significant metabolic perturbations in lipid metabolism (lipolysis), amino acid degradation, and the tricarboxylic acid (TCA) cycle in OSCC [121]. They documented elevated ketone body concentrations, suggesting enhanced lipolysis that may provide an alternative energy substrate. Moreover, these tumors predominantly relied on glycolysis and lactic acid fermentation, reflecting the Warburg effect [121,122]. Similarly, Yonezawa et al. used GC-MS to identify pronounced metabolic shifts, including alterations in glucose, methionine, ribulose, and ketoisoleucine levels, in recurrent HNSCC. They noted an inverse relationship between metabolite concentrations in serum and tumor tissue, notably lower serum amino acid concentrations (valine, tyrosine, serine, methionine) than elevated glycolytic metabolites [96].

Importantly, these metabolic signatures distinguished between recurrent and non-recurrent HNSCC patients [96]. Further research using NMR spectroscopy identified serum biomarkers, including glutamine, propionate, acetone, and choline, that effectively differentiated OSCC patients from healthy individuals, with high sensitivity and specificity. Additionally, a separate serum biomarker set (glutamine, acetone, acetate, choline) discriminated oral leukoplakia (OLK) from OSCC, underscoring the diagnostic potential of serum metabolomics across disease stages [123].

Urine is an advantageous biofluid for metabolomics, given its non-invasive collection and its ability to reflect systemic physiological and pathological changes, including malignancies [124,125]. Despite its advantages, research exploring urinary metabolomics studies in HNC remains limited. Xie et al. identified differentially expressed urinary metabolites (valine, 6-hydroxynicotinic acid, cysteine, and tyrosine) that successfully differentiated OSCC from healthy controls and OLK cases. Notably, valine and 6-hydroxynicotinic acid demonstrated high diagnostic accuracy, sensitivity, and specificity in distinguishing OSCC from controls, demonstrating promising potential for urinary metabolomics in HNC diagnostics [126]. Nonetheless, additional independent studies are imperative to validate these urinary biomarkers and expand their applicability for differentiating HNC patients from premalignant conditions and healthy controls.

#### 5.4.3. Cell and Tissue Metabolomics

While histopathological evaluation of tissue biopsies remains the gold standard for diagnosing head and neck cancer (HNC), this method can be subjective, as diagnostic criteria often vary between benign, premalignant, and malignant lesions. In contrast, metabolomic profiling generates a distinctive set of functional metabolites that objectively characterize disease phenotypes and are easier to interpret.

Bag et al. utilized 1H and 13C NMR studies to identify decreased choline levels and increased trimethylamine N-oxide (its metabolic degradation product) in oral squamous cell carcinoma (OSCC) biopsy tissues. They also noted unchanged lactate levels in OSCC compared to controls, suggesting the absence of a prominent “Warburg effect” in OSCC [107] The same research group later employed nano-LC-MALDI MS/MS to demonstrate alterations in lipid metabolites such as triglycerides, phosphatidylinositol, phosphatidylcholine, phosphatidylinositol bisphosphate, glycerophospholipid, and cytidine diphosphate diacylglycerol in oral submucous fibrosis (OSF) and OSCC samples. These findings indicated that changes in lipid metabolism were linked to membrane biogenesis in OSF and OSCC [108].

Conversely, Ogawa et al. used CE-TOF-MS to demonstrate increased glucose consumption and lactate production in OSCC tissues, indicating a prominent “Warburg effect.” Their study revealed significantly lower concentrations of glucose, 3-phosphoglycerate (3PG), and 2-phosphoglycerate (2PG) in OSCC tissues, along with higher lactate levels than in normal tissues [127]. Other researchers have also reported lower glucose levels in tumors relative to healthy controls [128].

Musharraf and colleagues identified 31 of 735 tissue metabolites that distinguished oral cancer from precancerous and control samples. Their results showed downregulated amino acid levels in OSCC tissues, suggesting increased energy metabolism and upregulation of biosynthetic pathways necessary for rapid cancer cell proliferation [108]. Additionally, oral metabolites associated with energy metabolism were elevated in HNSCC, as analyzed by LC-MS and GC-MS [86].

A study employing wax physisorption-based FTIR imaging of healthy keratinocytes and cancer cells demonstrated that methylene (CH_2_) and methyl group (CH_3_) stretching vibrations in the 3000–2800 cm^−1^ range could differentiate OSCC cells from healthy keratinocytes. The researchers concluded that this imaging technique could be used for early screening of oral cancer lesions [66]

Cancer cells exhibit remarkable metabolic plasticity, allowing them to adapt their energy sources by switching between glycolysis, lactic acid fermentation, and glutaminolysis [129,130,131,132]. In some cancer cell systems, this metabolic flexibility leads to a dependence on glutamine, with the oncogene Myc potentially orchestrating the expression of genes essential for glutamine breakdown [130]. Studies employing LC-MS in HNSCC cell lines revealed that glucose, rather than glutamine, was the primary energy source required for these cells to proliferate and survive [133]. However, cancer cells can adapt to different energy sources based on their genetic makeup and available substrates [132,133,134]. Recent research by Kamarajan et al. showed that high glutaminolysis activity is present in primary and metastatic HNSCC tissues and cells, characterized by elevated glutamate and reduced glutamine levels, as determined using advanced UPLC-MS/MS and GC-MS techniques [129]. Their study also demonstrated that glutamate is a key indicator of cancer metabolism, and its regulation via glutaminase cooperates with aldehyde dehydrogenase to facilitate cancer stemness [129]. These findings suggest that both glycolysis and glutaminolysis are crucial energy sources in HNSCC. However, glucose metabolism may be more critical for sustaining proliferation and survival, while glutamine/glutamate metabolism may drive aggressive phenotypic transitions, including the acquisition of stemness properties and metastatic potential.

An additional layer of complexity arises from anatomical subsite-specific and stage-dependent metabolic heterogeneity. OSCC, laryngeal, and oropharyngeal tumors differ substantially in stromal composition, hypoxic burden, and microbial exposure, all of which influence metabolic phenotypes. Early-stage lesions often exhibit partial metabolic reprogramming, whereas advanced and metastatic tumors display pronounced glutamine dependence, lipid remodeling, and redox adaptations.

Failure to account for these contextual variables likely contributes to inconsistencies across metabolomics studies and limits the reproducibility of biomarkers. Future investigations must stratify cohorts by HPV status, anatomical site, and disease stage to derive clinically actionable metabolic signatures.

## 6. Therapeutic Targeting of Metabolism in HNC

HNC is characterized by extensive metabolic reprogramming, enabling cancer cells to adapt to the complex tumor microenvironment and sustain aggressive proliferation. This metabolic plasticity is now recognized as a therapeutic vulnerability, and several key metabolic pathways are currently being investigated as potential drug targets [135].

### 6.1. Inhibitors of Glycolysis and Mitochondrial Function

HNC cells commonly upregulate glycolysis to thrive in hypoxic microenvironments. Targeting glycolytic enzymes, such as with 3-bromopyruvate (3-BrPA), an inhibitor of glyceraldehyde-3-phosphate dehydrogenase (GAPDH), has shown promising results by depleting ATP and inducing metabolic stress in tumor cells. Such inhibitors are progressing through early-phase clinical trials, underscoring their translational potential [136,137,138]. Mitochondria, as central regulators of cellular energy production and apoptosis, are also promising targets. Agents that disrupt mitochondrial function can elevate ROS levels and trigger apoptosis in cancer cells. Recent studies focus on molecules that selectively impair components of the mitochondrial respiratory chain, thereby sensitizing tumors to conventional therapies [139,140,141].

### 6.2. Targeting Amino Acid and Lipid Pathways

Altered lipid metabolism is another hallmark of HNC, with tumor cells exhibiting dysregulated lipid biosynthesis and utilization to support proliferation and survival. Therapeutic strategies aimed at inhibiting lipid metabolic pathways have shown potential to suppress tumor growth and induce apoptosis [142]. Similarly, amino acid metabolism, particularly glutaminolysis, is exploited by HNC cells to sustain anabolic processes and maintain redox balance. Inhibitors targeting these pathways are being explored as adjuncts to standard therapies.

### 6.3. Combining Metabolic Inhibitors with Radiotherapy or Immunotherapy

Combining metabolic inhibitors with established therapeutic modalities such as radiotherapy or immunotherapy is an emerging strategy to overcome resistance and enhance treatment efficacy. Modulating tumor metabolism can sensitize cancer cells to radiation and immune-mediated killing, yielding a synergistic therapeutic effect. This approach is designed to exploit metabolic vulnerabilities while concurrently targeting the proliferative and immune-evasive mechanisms of HNC [135] (Table 3).

However, translating these metabolic interventions into clinical practice requires careful navigation of potential adverse effects. Because core metabolic pathways such as glycolysis, glutaminolysis, and oxidative phosphorylation are essential for normal cellular homeostasis, systemic administration of metabolic inhibitors can induce significant off-target toxicities, including hepatotoxicity, fatigue, and gastrointestinal distress. In the context of HNSCC, combining metabolic therapies with radiation may exacerbate local tissue damage, potentially increasing the incidence and severity of radiation-induced mucositis. Furthermore, a paradoxical risk arises when combining these agents with immunotherapy: inhibiting nutrient uptake pathways to starve the tumor can inadvertently impair the activation and proliferation of healthy effector T cells, thereby counteracting the intended efficacy of immune checkpoint inhibitors.

To safely translate these findings into viable clinical practice, future strategies must focus on precision application and advanced formulations. Clinically, the metabolic biomarkers identified in biofluids can be used to stratify patients, ensuring that metabolic inhibitors are prescribed only to those whose tumors exhibit specific, measurable metabolic dependencies. Furthermore, developing localized, targeted drug-delivery systems, such as nanoparticle-based carriers, biodegradable hydrogels, or other functionalized biomaterials, can deliver metabolic inhibitors directly to the tumor microenvironment.

This targeted approach would maximize synergistic efficacy with radiotherapy or immunotherapy while shielding systemic immune cells and healthy tissues from off-target toxicity, representing a critical bridge between metabolomic discovery and personalized oncology.

## 7. Clinical Translation and Biomarker Development

The significance of metabolic biomarkers lies not only in their potential for early disease detection but also in their ability to inform personalized therapy and guide clinical decision-making, thereby improving patient outcomes [150,151]. The role of metabolic biomarkers extends to their function as prognostic indicators, offering insights into disease progression, therapeutic response, and overall prognosis. Despite their promise, implementing metabolic biomarkers in clinical practice faces substantial challenges. Variability in biomarker expression, a lack of standardized measurement protocols, and complexities in data interpretation hinder widespread adoption [12,152]. Addressing these challenges is critical to realizing the full potential of metabolic biomarkers to improve patient care and outcomes. As research progresses, the future of metabolic biomarker development and clinical translation holds great promise, particularly with advancements in personalized therapy and precision medicine. The integration of innovative analytical techniques and the emergence of digital biomarkers could further revolutionize the landscape, improve diagnostic and prognostic accuracy, and foster greater patient engagement in their healthcare journeys [153].

### 7.1. Metabolic Biomarkers for Early Detection and Prognosis

Metabolic biomarkers are increasingly recognized for their value in the early detection and prognosis of HNC. These biomarkers reflect disease-associated metabolic shifts and can be detected before clinical symptoms manifest, enabling timely intervention. Advanced analytical platforms have facilitated the identification of comprehensive biomarker panels capable of distinguishing early-stage disease and predicting disease progression (Table 4) [154,155]. In addition to diagnosis, metabolic biomarkers provide prognostic information, helping clinicians assess tumor aggressiveness, predict therapeutic outcomes, and stratify patients [156,157,158].

To establish true clinical utility, emerging metabolic biomarkers must be critically compared against existing diagnostic standards. Currently, HNSCC diagnosis and staging rely heavily on invasive tissue biopsies, histopathological grading, p16 immunohistochemistry (for HPV status), and structural/functional imaging (e.g., MRI, 18F-FDG PET/CT). While 18F-FDG PET/CT effectively captures the macroscopic Warburg effect (glucose uptake) to identify metastases, it lacks the molecular granularity to differentiate tumor progression from localized inflammation, leading to occasional false positives. Systemic metabolic biomarkers, such as plasma choline or lipid profiles, offer a distinct advantage here by providing real-time, molecular-level snapshots of tumor burden that could augment standard imaging and potentially distinguish true recurrence from radiation-induced tissue necrosis.

Furthermore, while scalpel biopsy remains the gold standard for definitive diagnosis, it is impractical for longitudinal screening of high-risk populations (e.g., heavy smokers or individuals with oral leukoplakia). In this context, salivary biomarkers (such as polyamines) do not aim to replace histopathology but rather serve as non-invasive triage tools. By identifying patients with high-risk metabolic signatures, clinicians can prioritize them for invasive biopsies, thereby reducing unnecessary procedures and accelerating the detection of malignant transformation.

Despite the growing number of metabolites reported in HNSCC metabolomic studies, not all biomarkers possess equal translational potential. Prioritization of candidate metabolites is therefore essential for advancing metabolomic findings toward clinical application. Metabolites consistently detected across multiple independent studies, analytical platforms, and biological matrices are stronger candidates for clinical translation. In particular, metabolites involved in polyamine metabolism, choline-containing phospholipids, glycolytic intermediates such as lactate, and alterations in amino acid metabolism have repeatedly emerged across HNSCC cohorts. Among these, systemic polyamine derivatives and choline-related metabolites appear especially promising because they can be detected in accessible biofluids, such as saliva or plasma, and exhibit relatively robust discriminatory performance across several studies. Nevertheless, most metabolomic biomarkers remain at early discovery or research validation stages, underscoring the need for standardized analytical methods, larger multicenter cohorts, and prospective validation before these candidates can be implemented in routine clinical practice.

### 7.2. Personalized Therapy Based on Metabolic Profiles

Personalized therapy guided by metabolic profiling represents an evolving paradigm in HNC management. By integrating metabolic, genetic, and clinical data, clinicians can tailor treatments to individual patient profiles, thereby maximizing therapeutic efficacy and minimizing toxicity [159]. Metabolic biomarkers guide the selection of optimal therapies and enable real-time monitoring of treatment response. Technological innovations, such as non-invasive biosensing devices and advanced metabolic assays, are accelerating the clinical adoption of this personalized approach. In oncology, repurposing dietary-derived bioactive compounds or existing pharmacological agents with established safety profiles is a promising strategy for rapid clinical translation [167].

### 7.3. Limitations and Challenges in Clinical Implementation

Despite significant progress, several barriers impede the clinical implementation of metabolic biomarkers and personalized therapy. Regulatory challenges, such as stringent patent laws and lengthy approval processes, complicate the commercialization and clinical adoption of novel biomarkers [168]. Technical barriers, such as high analytical costs, the lack of standardized protocols, and data integration complexities, further restrict widespread application [169,170]. Additionally, limited patient engagement and education remain obstacles, as direct communication between clinicians, pathologists, and patients is infrequent, potentially impeding patient understanding and acceptance of biomarker-driven care. Moreover, most research to date has focused on Western populations, with minimal validation in ethnically diverse or pediatric cohorts, underscoring the need for broader studies [171].

Looking ahead, the integration of metabolomics into precision medicine, advances in analytical technologies, and the development of digital biomarkers are expected to drive further progress. However, addressing technical, regulatory, and equity challenges will be essential to fully realize the potential of metabolic biomarkers and personalized therapy in routine HNC care.

## 8. Conclusions and Future Directions

This review highlights how dysregulated metabolism underpins the initiation and progression of HNSCC, from the well-established Warburg effect to emerging one-carbon and lipid-ferroptosis pathways. By examining advances in chromatography, mass spectrometry, NMR, and spatial metabolomics, we highlighted not only the depth of molecular insight now possible but also the practical challenges, including batch variability, limited cohort sizes, and cross-platform standardization, that continue to hinder broad clinical application.

Although numerous metabolomic studies have reported potential biomarkers for HNSCC, only a limited number have progressed beyond the discovery phase. Most studies remain exploratory and use relatively small patient cohorts, which can limit reproducibility across independent datasets. Consequently, translating metabolomic biomarkers into clinical practice requires a structured framework encompassing biomarker discovery, analytical validation, clinical validation, and demonstration of clinical utility.

Several metabolite classes, including amino acids, lipid metabolites, and glycolytic intermediates, have been reported to be significantly altered in HNSCC tissues and biofluids, such as saliva and plasma. However, the diagnostic performance of these metabolites varies considerably across studies, with reported sensitivity and specificity values varying by analytical platform, sample type, and cohort characteristics.

A major barrier to clinical translation remains the lack of standardized workflows for sample preparation, data acquisition, and metabolite annotation. Variability across analytical platforms and bioinformatic pipelines has contributed to inconsistencies between studies. Large multicenter validation studies and standardized analytical protocols will therefore be essential to establish reproducible metabolomic biomarkers.

Looking forward, integrating single-cell metabolomics with high-resolution imaging promises to resolve intratumor heterogeneity in unprecedented detail, while machine-learning approaches offer a path to translate complex spectral data into actionable biomarkers. Multi-omics studies that couple metabolic profiling with genomics and proteomics will be essential for constructing a holistic portrait of tumor biology and for identifying patient-specific metabolic vulnerabilities.

Currently, the clinical diagnosis and staging of HNSCC rely on imaging techniques such as CT, MRI, and positron emission tomography (PET), together with histopathological evaluation of tumor biopsies and molecular testing such as HPV status assessment. While these approaches provide critical anatomical and molecular information, they do not directly capture metabolic alterations associated with tumor biology. Metabolomic profiling, therefore, represents a complementary strategy that may improve early detection and monitoring; however, rigorous validation and standardization are required before metabolomics-derived biomarkers can be integrated into routine clinical practice.

Ultimately, realizing the clinical promise of metabolomics will require close collaboration between basic researchers, clinicians, and bioinformaticians. Standardized protocols, larger prospective cohorts, and shared data repositories will be vital for reproducing findings and for moving candidate biomarkers through the pipeline to diagnostic assays or therapeutic stratification tools. We envision a future in which metabolomic signatures guide personalized treatment decisions, whether by predicting response to immunotherapy, monitoring minimal residual disease through plasma or saliva, or uncovering novel metabolic targets for small-molecule inhibitors.

By embracing technological innovation alongside rigorous methodological standards, the field can transform metabolic discoveries into tangible benefits for patients with HNSCC and, in doing so, establish a template for metabolomics-driven precision oncology across a broad spectrum of tumor types.

### Bridging Metabolomic Discovery to Clinical Application in HNSCC

Although metabolomic investigations in HNSCC have generated a large body of data describing altered metabolites and pathways, the strength of evidence and translational readiness of these findings vary considerably. Most studies differ in cohort size, analytical platforms, biological matrices, and clinical annotations, making it essential to distinguish exploratory associations from biomarkers with genuine clinical potential. A structured evaluation of metabolomic evidence, rather than reliance on statistical significance alone, is therefore required to bridge the gap between discovery and clinical application.

At the lowest level of evidence, many reported biomarkers originate from small, single-center, cross-sectional studies that use untargeted metabolomics. These findings are often biologically informative and valuable for hypothesis generation; however, they frequently lack independent replication and are susceptible to confounding factors such as diet, tobacco exposure, inflammation, microbiome composition, and treatment status. Consequently, such metabolite associations should not be interpreted as clinically actionable biomarkers but rather as exploratory signals requiring further validation.

A higher level of evidence is represented by metabolites and pathways that recur across multiple independent studies, analytical platforms, or biological matrices. Reproducible alterations in glycolysis, amino acid metabolism, choline-containing compounds, lipid metabolism, and polyamine pathways consistently emerge across HNSCC cohorts. While these findings demonstrate biological plausibility and robustness, most lack external or prospective validation and therefore remain preclinical. Importantly, reproducibility alone does not guarantee clinical applicability in the absence of standardized assays and defined clinical endpoints.

More advanced evidence is observed when metabolomic alterations correlate with clinically meaningful outcomes such as tumor stage, prognosis, treatment response, or human papillomavirus (HPV) status. These biomarkers are typically supported by moderate-to-large patient cohorts and provide a stronger translational rationale. Nevertheless, much of the available evidence remains retrospective or single-center, and assay standardization and multicenter validation are often lacking, limiting immediate clinical deployment.

Only a limited number of metabolomic biomarkers are ready for clinical use. These candidates are characterized by validation across independent or multicenter cohorts using targeted, quantitative analytical methods and predefined clinical endpoints. In HNSCC, systemic polyamine signatures and choline-related metabolic profiles currently represent the most promising examples approaching this level, although further prospective validation is still required. This scarcity underscores the current translational gap in HNSCC metabolomics and highlights the need for coordinated validation efforts.

A critical challenge in the field is the frequent conflation of metabolic associations with clinically actionable biomarkers. Many metabolite changes reflect tumor burden, hypoxia, immune activation, or treatment-induced metabolic stress rather than tumor-specific biology. For a metabolomic biomarker to be clinically useful, it must demonstrate consistent performance across independent cohorts, robust analytical reproducibility using standardized assays, specificity beyond systemic confounders, and incremental value over existing clinical or molecular markers. Without meeting these criteria, metabolomic findings should be interpreted primarily as insights into tumor biology rather than diagnostic or prognostic tools.

Translating metabolomic discoveries into clinically applicable biomarkers in HNSCC requires a structured and stepwise pipeline. Initial discovery should be performed using untargeted metabolomics in well-phenotyped patient cohorts. Candidate metabolites must then be prioritized based on biological relevance, recurrence across studies, and association with tumor progression, immune modulation, or therapeutic response. This should be followed by analytical validation through the development of targeted, quantitative assays with standardized protocols and inter-laboratory reproducibility. Subsequent clinical validation in independent and preferably multicenter cohorts, ideally using prospective study designs with predefined clinical endpoints, is essential. Ultimately, successful biomarkers must be integrated with existing clinical, pathological, and molecular parameters and undergo regulatory qualification to demonstrate clinical utility.

At present, the majority of metabolomic biomarkers reported in HNSCC remain at early exploratory or reproducibility stages, emphasizing the urgent need for standardized analytical pipelines, multicenter collaboration, and prospective validation. Addressing these challenges will be critical to realizing the full potential of metabolomics for precision oncology in HNSCC. In addition to evaluating the strength of evidence, it is equally important to prioritize metabolomic biomarkers based on their translational potential and clinical applicability.

**Figure 4:**
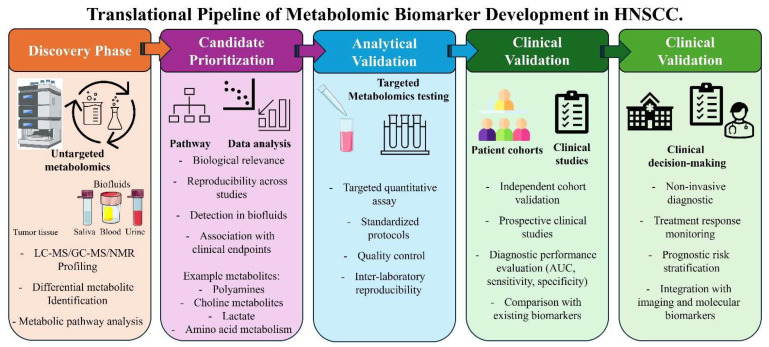
Translational Pipeline of Metabolomic Biomarker Development in HNSCC.

To further clarify translational readiness, metabolomic biomarkers reported in HNSCC can be broadly stratified into three categories based on the level of supporting evidence: exploratory candidates, reproducible research biomarkers, and clinically translatable markers. The sequential steps required to translate metabolomic discoveries into clinically actionable biomarkers are summarized in Figure 4. Exploratory candidates typically arise from single-center untargeted metabolomics studies and include diverse metabolites associated with glycolysis, amino acid metabolism, lipid remodeling, and ketone body pathways. While these findings provide important biological insights, their clinical interpretability remains limited due to small cohort sizes, heterogeneous analytical platforms, and the absence of independent validation. A second tier includes metabolites that have demonstrated reproducibility across multiple studies or sample types, such as alterations in choline-containing compounds, polyamine metabolism, aromatic amino acids, and glutamine-glutamate pathways. These biomarkers exhibit stronger biological plausibility and recurrence across independent datasets; however, most remain at the research validation stage due to the lack of standardized assays and multicenter prospective validation. Only a small subset of metabolites, particularly systemic polyamine derivatives (e.g., N1-acetylspermine) and choline-related lipid metabolites, show emerging potential for clinical translation because they have been detected consistently in accessible biofluids such as saliva or plasma and demonstrate relatively robust discriminatory performance across independent cohorts.

Importantly, the clinical value of metabolomic biomarkers must be evaluated relative to existing diagnostic and prognostic tools used in HNSCC management. Current clinical practice relies primarily on imaging modalities such as CT, MRI, and PET, combined with histopathological evaluation and HPV molecular testing, which provide anatomical and molecular information but offer limited insight into tumor metabolic activity. Metabolomic biomarkers, therefore, hold promise as complementary tools capable of capturing dynamic metabolic alterations associated with tumor progression, treatment response, and immune-metabolic interactions. For example, biofluid-based metabolite panels derived from saliva or plasma could enable non-invasive early detection, longitudinal disease monitoring, or treatment response assessment, functions that are not readily achieved with conventional tissue biopsy or imaging alone. Nevertheless, to demonstrate true clinical utility, metabolomic biomarkers must show incremental diagnostic or prognostic value beyond current standards, including improved sensitivity for early-stage disease, the ability to predict therapeutic response, or reliable monitoring of minimal residual disease. Achieving this goal will require rigorous prospective validation studies, standardized analytical pipelines, and integration of metabolomic markers with established clinical and molecular parameters within multi-modal diagnostic frameworks.

## Figures and Tables

**Table 1:** Advantages and disadvantages of metabolite identification methods.

Methods	Advantages	Disadvantages	References
HPLC	➢ Suitable for a variety of analyte sample kinds Accurate and highly repeatable quantitative analysis	➢ Inadequate universal detector ➢ Inferior separation efficiency compared to capillary GC	[66]
NMR	➢ Non-destructive, high resolution, and very reproducible ➢ Identifies all metabolite simultaneously ➢ No need of sample derivatization	➢ Low sensitivity (concentration of µM to mM) ➢ Multiple peaks for each component ➢ Limited library usage as a result of the intricate matrix	[67]
GC	➢ High resolution, reliable, and relatively simple ➢ Highly accurate quantitative analysis ➢ High sensitivity, detecting down to 100 ppm ➢ Quick analysis	➢ Only volatile samples are allowed. ➢ Unable to identify compounds that are thermally labile ➢ MS is needed to confirm peak identity. ➢ Destructive detector	[68]
MS	➢ Distinct spectra that are simple to interpret ➢ Molecular weight measures isotopic ratios and can be calculated from a very small sample. ➢ Compared to previous methods, detection limits are three times higher.	➢ Incapable of differentiating between compounds with identical charge-to-mass ratios ➢ Costs two to three times as much as other instruments ➢ There could be interference effects. ➢ Drift might reach 5% to 10% per hour.	[69]
GC-MS	➢ Extremely robust and sensitive ➢ Fit for analyzing hydrophobic substances and combinations with low molecular weights ➢ Direct analysis of volatile chemicals is possible ➢ Provides a wide linear range	➢ Incapable of identifying thermo-unstable and non-volatile substances ➢ Derivatization can be necessary, and it may obscure the outcome.	[68]
LC-MS	➢ It can be used to analyze moderately polar molecules with molecular weights of low, moderate, or high. ➢ Possible to analyse thermo-unstable molecules. ➢ No need of sample derivatization process ➢ Extremely automated, adaptable, and sensitive	➢ Various kinds of adducts can be formed. ➢ Time-consuming and susceptible to substances that interfere ➢ Limited to the mass range ➢ Fragmentation patterns are difficult to replicate.	[68]
CE-MS	➢ Very sensitive, quick, and inexpensive ➢ Low sample volumes are sufficient ➢ Multidimensionality and automation	➢ Unable to identify proteins greater than 20 kDa	[69,70]

**Table 3:** Summary of Inhibitors of Metabolic Pathways in HNSCC.

Inhibitor/Drug	Target/Pathway	Mechanism/Action	Immune/Tumor Effect	Clinical Status/Trial	Reference
Ganetespib	HSP90/Glycolysis	Inhibits HSP90, suppresses glycolytic enzymes (PKM2), reduces glycolytic flux	Enhances T cell infiltration, radio sensitization	NCT02334319	[135,143]
2-Deoxy-D-glucose (2-DG)	HK2/Glycolysis	Glucose analog, inhibits hexokinase, blocks glycolysis, reduces ATP	Inhibits proliferation, enhances radiosensitivity	Preclinical/Early Clinical	[133]
Rapamycin	mTOR/Glycolysis	Inhibits mTOR signaling, reduces PKM2, PDK1, HIF-1α, LDH, GLUT1	Reduces lactate, enhances immune-mediated tumor clearance	NCT01195922	[144]
Metformin	Mitochondrial Complex I/AMPK/mTOR	Inhibits complex I, activates AMPK, inhibits mTOR, reduces OXPHOS/glycolysis	Inhibits proliferation, enhances antitumor immunity, synergy with ICI	Preclinical	[145]
Atovaquone	Mitochondrial Complex III	Inhibits complex III, reduces oxygen consumption rate (OCR), increases ROS	Suppresses tumor growth, induces apoptosis	Preclinical/Early Clinical	[146]
PFK15	PFKFB3/Glycolysis	Inhibits PFKFB3, blocks glycolysis and lactate production	Suppresses proliferation and metastasis	Preclinical	[147]
Dichloroacetate (DCA)	PDK/Mitochondrial Metabolism	Inhibits pyruvate dehydrogenase kinase, shifts metabolism to oxidative phosphorylation	Reverses Warburg effect, potential for immune modulation	Preclinical/Other Cancers	[148]
CB-839 (Telaglenastat)	GLS1/Glutamine metabolism	Inhibits glutaminase, blocks conversion of glutamine to glutamate	Reduces PMN-MDSC recruitment, enhances antitumor immunity	Preclinical	[135,149]
Epacadostat, Pembrolizumab, BMS986205, IDOi	IDO1/Kynurenine pathway	Inhibit indoleamine 2,3-dioxygenase (IDO1), block tryptophan metabolism	Reduce immunosuppression, enhance ICI response	NCT03358472; NCT03854032	[141,142]
Etomoxir	CPT1/Fatty acid oxidation	Inhibits carnitine palmitoyltransferase 1, blocks fatty acid β-oxidation	Reverses M2 macrophage polarization, potential antitumor immunity	Preclinical	[135]
Orlistat, TVB-3166, C75, Triclosan	FASN/Fatty acid synthesis	Inhibit fatty acid synthase, block de novo lipogenesis	Reduce proliferation, migration, and metastasis in OCSCC/HNSCC models	Preclinical	[143]

**Table 4:** Overview of Clinically Relevant Metabolic Biomarkers in HNSCC.

Sample Type	Biomarker Name(s)	Analytical Method	Clinical Relevance	Reference
Tissue	Spermine synthase	LC-MS	Significant biomarker identified in HNSCC patients	[159]
Serum	Glutamate, Glucose and One carbon metabolites	NMR	Distress and pain significantly affected the serum lipids and one carbon metabolites in HNSCC patients	[160]
Serum	Glutamine, Glutamate, Citrulline, Ornithine	LC-MS	Altered amino acid metabolism; early detection and prognosis in HNSCC patients	[161]
Tumor Tissue and Adjacent Normal	Kynurenine	LC-MS (UHPLC-Q-Exactive MS)	Significant dysregulation in amino acid metabolism, especially elevated kynurenine. Kyn/Siglec-15 axis identified as a promising immunometabolism therapeutic target in HNSCC	[162]
Plasma	Acetyl Co-A and Fatty acid synthesis metabolites	LC-MS/MS	Plasma metabolites were significantly upregulated in HNSCC patient cohorts	[163]
Tissue/Serum	Choline, Carnitine, Lipid metabolites	MS, NMR	Lipid metabolism dysregulation linked to tumor progression and survival in HNC patients	[142]
Serum	Phenylalanine, Tyrosine, Tryptophan	LC-MS	Altered aromatic amino acid metabolism; immune evasion and prognosis in HNC patients	[164]
Serum	Arginine and proline	UHPLC-Q-Orbitrap HRMS	The prognosis-related genes and metabolites in HNSCC were mainly enriched in the purine metabolism pathway	[140]
Saliva	Fucose, proline, 1,2-propanediol	NMR	Point-of-care platforms for HNSCC	[88]
Serum	Succinate	LC-MS/MS	Oncometabolite accumulation associated with HIF-1α activation and treatment resistance	[65]
Serum	β-hydroxybutyrate, acetone, and acetoacetate	LC-MS	Increased levels of ketone bodies in HNSCC patients	[165]
Saliva	N1-acetylspermine	LC-MS/MS	Early diagnosis in HNC patients	[90]
Urine	Volatile organic metabolites	GC-MS	VOMs were significantly upregulated in all the HNSCC patients	[166]
Oral wash and Tissue	β-alanine,α-hydroxyisovalerate, tryptophan, and hexanoylcarnitine; 2-hydroxyglutarate (2-HG) and 3-GMP	LC-MS/MS, GC-MS	Small molecules related to energy metabolism were significantly elevated in HNSCC patients compared to controls	[101]

## Data Availability

No new data were created or analyzed in this study. Data sharing is not applicable to this article.
